# Untargeted Urinary Metabolomics and Children’s Exposure to Secondhand Smoke: The Influence of Individual Differences

**DOI:** 10.3390/ijerph18020710

**Published:** 2021-01-15

**Authors:** Huiwei Zhu, Abu S. Abdullah, Jingyi He, Jianxiong Xi, Yimeng Mao, Yitian Feng, Qianyi Xiao, Pinpin Zheng

**Affiliations:** 1 Department of Preventive Medicine and Health Education, School of Public Health, Fudan University, Shanghai 200032, China; 18211020128@fudan.edu.cn (H.Z.); 16211020052@fudan.edu.cn (J.H.); 17211020102@fudan.edu.cn (J.X.); 18211020031@fudan.edu.cn (Y.M.); 17211020028@fudan.edu.cn (Y.F.); zpinpin@shmu.edu.cn (P.Z.); 2 Key Lab of Public Health Safety of the Ministry of Education and Key Lab of Health Technology Assessment of the Ministry of Health, School of Public Health, Fudan University, Shanghai 200032, China; 3 Global Health Research Center, Duke Kunshan University, Kunshan 215347, China; 4 Duke Global Health Institute, Duke University, Durham, NC 27710, USA; 5 Department of General Internal Medicine, School of Medicine, Boston University Medical Center, Boston, MA 02118, USA

**Keywords:** secondhand smoke, metabolomics, untargeted, children, cotinine

## Abstract

Children’s exposure to secondhand smoke (SHS) is a severe public health problem. There is still a lack of evidence regarding panoramic changes in children’s urinary metabolites induced by their involuntary exposure to SHS, and few studies have considered individual differences. This study aims to clarify the SHS-induced changes in urinary metabolites in preschool children by using cross-sectional and longitudinal metabolomics analyses. Urinary metabolites were quantified by using untargeted ultra high-performance liquid chromatography-mass spectrometry (UPLC^(c)^-MS/MS). Urine cotinine-measured SHS exposure was examined to determine the exposure level. A cross-sectional study including 17 children in a low-exposure group, 17 in a medium-exposure group, and 17 in a high-exposure group was first conducted. Then, a before–after study in the cohort of children was carried out before and two months after smoking-cessation intervention for family smokers. A total of 43 metabolites were discovered to be related to SHS exposure in children in the cross-sectional analysis (false discovery rate (FDR) corrected *p* < 0.05, variable importance in the projection (VIP) > 1.0). Only three metabolites were confirmed to be positively associated with children’s exposure to SHS (FDR corrected *p* < 0.05) in a follow-up longitudinal analysis, including kynurenine, tyrosyl-tryptophan, and 1-(3-pyridinyl)-1,4-butanediol, the latter of which belongs to carbonyl compounds, peptides, and pyridines. Kyoto Encyclopedia of Genes and Genomes (KEGG) enrichment analysis indicated that 1-(3-pyridinyl)-1,4-butanediol and kynurenine were significantly enriched in xenobiotic metabolism by cytochrome P450 (*p* = 0.040) and tryptophan metabolism (*p* = 0.030), respectively. These findings provide new insights into the pathophysiological mechanism of SHS and indicate the influence of individual differences in SHS-induced changes in urinary metabolites in children.

## 1. Introduction

Children’s exposure to secondhand smoke (SHS) is a major public health problem, with a prevalence of 40% worldwide and 55.9% in Southeast Asia [1,2]. In China, 76.5% of respondents from 2124 families in six counties reported smoking in front of children [3]. Compared to adults, children are more likely to be affected by exposure to SHS at the same concentration since they inhale air at relatively higher ventilation rates [4]. SHS exposure results in serious health outcomes among children aged 4–11, including asthma, pneumonia, middle ear disease, and impaired endothelial function, and predicts long-term atherosclerotic disease progression and cardiovascular event rates [5,6,7,8,9], as well as thousands of avoidable hospitalizations [10]. However, few studies have focused on SHS exposure in preschool children aged < 5 years. These provocative findings warrant systematic investigations and early biomarker exploration for SHS-induced health problems in children.

Tobacco smoke contains more than 4000 kinds of constituents, including nicotine, tar, carbonic monoxide, polycyclic aromatic hydrocarbons, and heavy metals [11]. Nicotine is the primary component of tobacco and tobacco smoke [12]. Cotinine, which is a major metabolite of nicotine, has biological stability with a half-life of 20–30 h, while nicotine has a half-life of 2 h [13]. Urinary cotinine concentrations in children have been proven to indicate the degree of SHS exposure [14,15,16,17,18]. Studies have shown that exposure to tobacco smoke and nicotine during in utero and postnatal life impairs lung development, increases the susceptibility to lower respiratory tract infections, increases the prevalence of wheezing, and exacerbates respiratory symptoms in children with chronic lung diseases [19,20]. However, although urinary cotinine concentrations can produce a global profile of SHS exposure, they cannot provide specific clues regarding the functional pathway affected by SHS exposure and the pathophysiological mechanisms of SHS.

Metabolomics has emerged as a powerful tool for understanding metabolic changes in response to pathophysiological conditions or environmental exposures, providing an opportunity to identify biomarkers of exposure to tobacco smoke and markers that reflect host-related metabolic adaptations [21]. Several studies have indicated the value of metabolites in unraveling the biological mechanisms of the association of tobacco exposure with asthma [22], lung cancer [23], bladder cancer [24], and perinatal adverse outcomes [25]. Previous studies are largely focused on targeted metabolites caused by SHS [26,27]. In recent years, Gu et al. examined associations between cigarette smoking and metabolites using an untargeted metabolomics approach and identified 25 metabolites associated with smoking behaviors [28]. However, there is still a lack of knowledge on panoramic changes in urinary metabolites in response to children’s exposure to SHS, and few studies have considered individual differences, such as heredity and the physiological status. In the present study, we used an untargeted metabolomics approach to explore the panoramic changes in urinary metabolites associated with children’s exposure to SHS. In addition, we combined the cross-sectional and longitudinal metabolomics analyses to exclude individual differences. Specifically, differentially expressed urinary metabolites were first discovered at different levels of the SHS-exposure group in cross-sectional analyses. Then, a before–after study of the cohort of children was carried out before and two months after the smoking-cessation intervention for family smokers and the implicated urinary metabolites in cross-sectional analyses were confirmed in longitudinal analyses.

## 2. Materials and Methods

### 2.1. Subject Recruitment and Sample Collection

Subject recruitment was carried out in the Hetou and Luoyang village communities, Taizhou city, Zhejiang province, China, from 15 March 2018 to 31 March 2018. The cluster sampling method was used to select the Hetou and Luoyang village communities. Smokers were identified using the health records of community health service stations. Study coordinators went to each smoking family to describe the study. Smoking families were enrolled if they met the following criteria: (1) Smokers reported having children aged 2–5 years and smoking at home and in front of children; (2) caregivers of children were willing to provide the children’s urine samples; and (3) both the smokers and the caregivers of children provided written informed consent. At the baseline, data collection was performed via an in-person questionnaire survey administered to smokers and caregivers. Data included the smoking site/venues, the smoking frequency in the past week, and the average daily cigarette amount in the past week for smokers, and the health condition of and recent respiratory symptoms among children.

The urine samples from each child were collected in sterile fecal collection containers at home or at a community health service station and then placed in a portable refrigeration apparatus (−20 °C) immediately after sampling. Upon receipt, the urine samples were immediately frozen and stored at −80 °C until analysis.

### 2.2. Ethics Statement

This study was approved by the ethics committee of Duke Kunshan University (IRB No: 2016ABDU003). Written informed consent was obtained from each smoker and caregiver of the child.

### 2.3. Follow-Up Interview

From 15 May 2018 to 15 July 2018, we conducted smoking-cessation interventions for smokers, including health education counseling delivered by community health workers in five different sessions (two in-person and three via-telephone sessions). The counseling covered hazards about smoking and SHS exposure, tips on quitting smoking and overcoming withdrawal syndrome, how to prevent relapse, and how to ensure a smoke-free home. Follow-up interviews and assessments were conducted at 2 months after the intervention for smokers and caregivers of children. Urine samples were also collected at the follow-up.

### 2.4. Cotinine Measurements, Exposure Level Stratification, and Quality Control

Urine cotinine was analyzed using a liquid chromatography tandem mass spectrometry (LC-MS) method. Urine cotinine levels were reported in nanograms per milliliter and standardized per milligram of creatinine. Cotinine concentrations below the limit of detection (LOD, 0.5 ng/mL) were considered unobservable and inaccurate [13,29], and were excluded. For baseline cross-sectional analysis, the children were stratified into three subgroups by cotinine concentration: Baseline low exposure (BL, 0.5–2 ng/mL); baseline medium exposure (BM, 2–10 ng/mL); and baseline high exposure (BH, 10–40 ng/mL) [18,30]. For longitudinal analysis, changes in the cotinine concentration from each child were assessed. The children at 2 months after intervention were stratified into two groups based on changes in the cotinine concentration: An intervention-no-changed (INC) group (children with a change in the cotinine concentration within 0.5 ng/mL) and intervention-declined (ID) group (children with a declined cotinine concentration of more than 2 ng/mL).

### 2.5. Definition of SHS Exposure in This Study

SHS exposure was determined by smokers’ self-reports and by measuring the children’s urinary cotinine concentrations. Positive SHS exposure was defined when both the self-reported SHS exposure in children by smokers and urine cotinine-measured SHS exposure were positive.

Children were excluded if they met the following criteria: (1) Lost to follow-up; (2) those with missing laboratory measurements of the urinary cotinine concentration; and (3) those with a cotinine concentration at the boundary value according to the cutoff value of group stratification.

### 2.6. UPLC^(c)^-MS/MS-Based Urine Metabolomics

Urine samples (100 μL) were added to 300 μL methanol-water (2:1, *v*/*v*) [31]. Each sample was homogenized for 1 min, ultrasonically extracted on ice for 10 min, stored at −20 °C for 30 min, and then centrifuged at 13,000 rpm for 15 min at 4 °C. Next, 200 μL supernatant was transferred to a new vial for LC-MS/MS analysis. A mixture of all extraction aliquots was used as a quality control (QC) sample for LC-MS/MS analysis. LC-MS/MS-based urinary metabolic profiling was performed on an Ethylene Bridged Hybrid C18 column (100 mm id × 2.1 mm, 1.7 μm internal diameter, Waters Corp., Milford, MA, USA) coupled with a Triple TOF TM 5600 mass spectrometer system (AB SICEX, Framingham, MA, USA). The EBH C18 column was maintained at 45 °C for chromatographic separation. The prepared sample was injected and maintained at 4 °C for analysis. Samples were eluted using solvent A (aqueous formic acid (0.1% (*v*/*v*) formic acid) and solvent B (acetonitrile (0.1% (*v*/*v*) formic acid) at a flow rate of 0.40 mL/min. The separation was achieved with the following elution gradient: 1% B over 0–1 min; 1% B-20% B over 1–5.5 min; 20% B-30% B over 5.5–6 min; 30% B-35% B over 6–8.5 min; 35% B-70% B over 8.5–10.5 min; 70% B-100% B over 10.5–11 min; and the composition was held at 100% B for 2 min, and then 13–13.1 min, 100% B to 1% B, and 13.1–15 min holding at 1% B. The MS signal acquisition was performed in positive and negative ion scanning modes. To obtain information regarding the system repeatability, QC samples were injected at regular intervals (every six analytical samples) throughout the analytical run [32].

### 2.7. Bioinformatic and Statistical Analyses

UPLC^(c)^-MS/MS raw data were processed using Progenesis QI software (Waters Corp, Milford, MA, USA), and a data matrix containing the retention time, mass-to-charge ratio, and peak intensity was obtained. The data matrix was preprocessed as follows: By (1) retaining variables with >50% of nonzero values in all samples [33]; (2) filling the missing values by half of the minimum value in the original matrix to decrease the false positive results [34]; (3) normalizing the total peaks and deleting variables with Relative Standard Deviation ≥ 30% in the QC samples; and (4) performing a log10 conversion, resulting in a data matrix that was used for subsequent analysis. The mass spectra of these metabolic features were identified by using the accurate mass, MS/MS fragment spectra, and isotope ratio difference by searching in reliable biochemical databases such as the Human Metabolome Database (HMDB) (http://www.hmdb.ca/) and Metlin database (https://metlin.scripps.edu/). Concretely, the mass tolerance between the measured *m*/*z* values and the exact mass of the components of interest was ±10 ppm. For metabolites having MS/MS confirmation, only the ones with MS/MS fragment scores above 30 were considered as having been confidently identified; otherwise, metabolites only had tentative assignments. The positive and negative data were combined and imported into the SIMCA-P + 14.0 software package (Umetrics, Umea, Sweden). Data analysis was performed on the Majorbio I-Sanger Cloud platform (www.i-sanger.com).

Orthogonal partial least-squares discriminant analysis (OPLS-DA) was performed using the ropls package in R to visually discriminate between BL and BH groups, as well as between BL and BM groups at the baseline. This approach aims to maximize the covariance between the outcome and matrix of metabolites by projecting both to linear subspaces of the original variables [35,36]. Validation for OPLS-DA models was conducted in a seven-fold cross-validation process, and model overfitting was examined in a 200-fold permutation test. R^2^Y and Q^2^ were used to evaluate the goodness-of-fit and predictive ability of each model. Through an analysis of OPLS-DA loadings, taking the BL group as a reference, an independent-sample hypothesis test was used for the BH and BL groups, as well as for the BM and BL groups, in the cross-sectional study. The differential metabolites were identified with a variable importance in the projection (VIP) of greater than 1.0 and false discovery rate (FDR) corrected *p* values of less than 0.05 (Student’s t-test using the stats package in R). A cluster heatmap of the metabolites identified in this process was constructed using the Pheatmap package in R. The paired-sample hypothesis test (paired t-test using the stats package in R) was used to verify the differential urinary metabolites through a comparison of before intervention (pre-intervention) and after intervention (post-intervention) paired-samples from each child in the ID and INC group in the longitudinal study. Significant differences were considered when results were below an FDR threshold of 0.05. The Kyoto Encyclopedia of Genes and Genomes (KEGG) pathway database and HMDB database were also used to uncover the predicted molecular pathways and biological functions of the metabolites. KEGG enrichment analysis of common target metabolites was performed using Fisher’s exact test to obtain the significantly enriched pathways [37]. All of these analyses were performed using R version 3.6.1 (R Foundation for Statistical Computing, Vienna, Austria). and the Majorbio I-Sanger Cloud platform (www.i-sanger.com).

Analyses of demographic data were carried out using SPSS software. (version 23.0, IBM, Chicago, USA). Continuous variables were described with the mean and standard deviation (SD), and were compared with regards to the three baseline SHS-exposure level groups by using analysis of variance (ANOVA) and the pre-intervention and post-intervention groups by using a paired Student’s *t*-test. Categorical variables were described with numbers and percentages, and were analyzed by Fisher’s exact test in terms of the three baseline SHS-exposure level groups and McNemar’s test for the pre-intervention and post-intervention groups. All tests for significance were two-sided, and *p*-values < 0.05 were considered significant.

## 3. Results

### 3.1. Study Subjects at the Baseline

**Figure 1:**
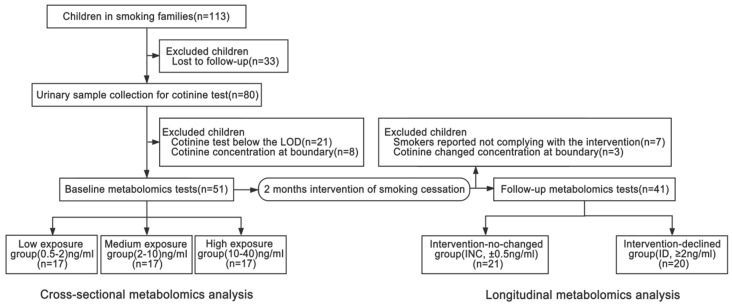
Flowchart of included children at the baseline and follow-up. LOD, limit of detection.

Figure 1 details the recruitment process. Among the 113 enrolled children in a smoking family at the baseline, 33 were lost to follow-up, 21 had cotinine concentrations below the LOD, and 8 had cotinine concentrations at the boundary value according to the cutoff value of group stratification. Therefore, a total of 51 preschool children aged 2–5 years who were in a smoking family were included for analysis.

Table 1 lists the subjects’ characteristics at the baseline. For family smokers, the average number of smoking days in the past week was 6.88 (SD 0.62), and the average daily cigarette amount in the past week was 19.70 (SD 10.20). There was no significant difference in the smoking frequency or smoking amount among with different SHS exposure groups defined by cotinine concentration. Among the 51 children, 88% were healthy, 18% had throat irritation or pain, 18% exhibited wheezing, and 24% had experienced nasal obstruction in the past six months, as reported by their caregivers. There was no significant difference in these symptoms among the different SHS exposure groups.

### 3.2. Cross-Sectional Metabolomics Analysis at the Baseline

**Figure 2:**
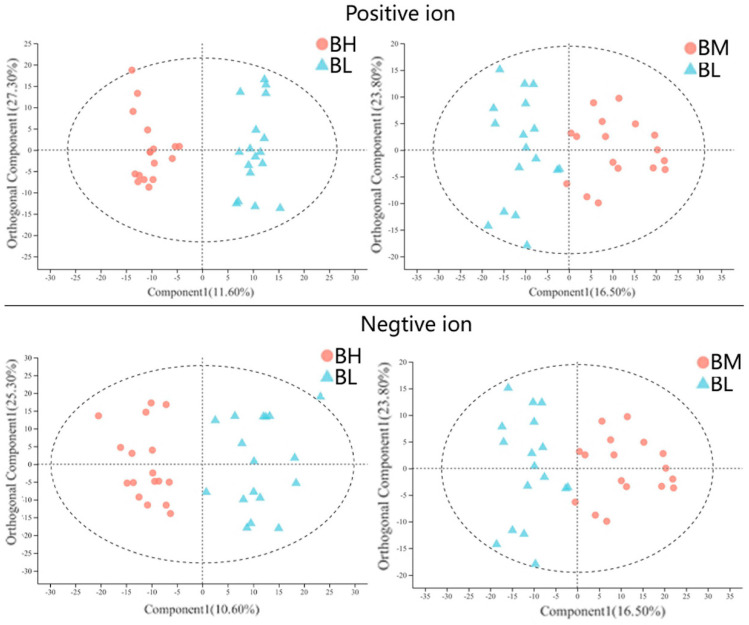
Orthogonal partial least-squares discriminant analysis (OPLS-DA) score plots for BH and BL groups, as well as for BM and BL groups, in positive-ion and negative-ion modes. Abbreviations: BH, high-level SHS exposure at the baseline; BM, medium-level SHS exposure at the baseline; and BL, low-level SHS exposure at the baseline.

**Figure 3:**
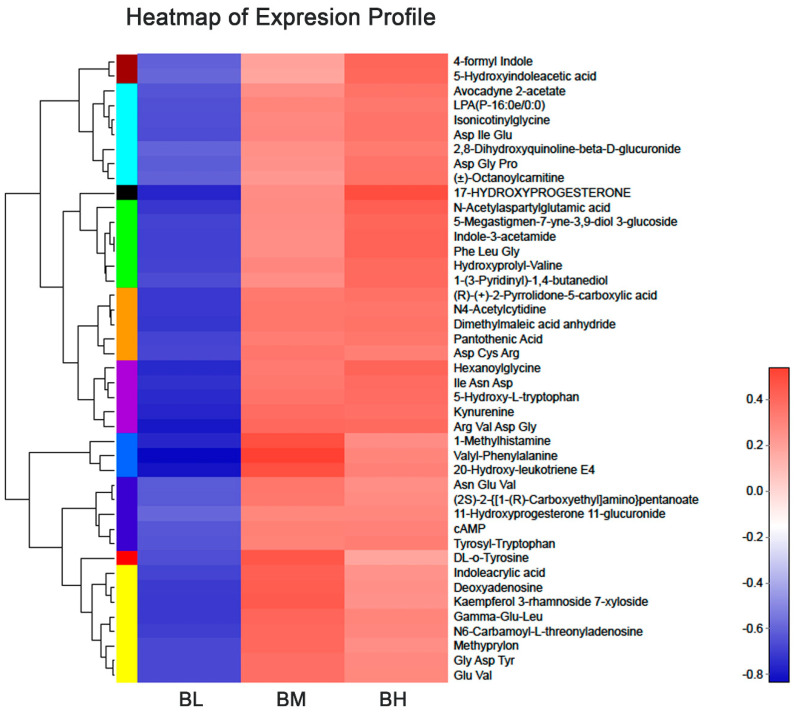
Heatmap of the levels of differentially expressed metabolites in each group at the baseline.

The OPLS-DA score plot revealed a clear separation between high-exposure (BH) and low-exposure (BL) groups, as well as between medium-exposure (BM) and BL groups (Figure 2). Seven-fold cross-validations R^2^ Y and Q^2^ indicated a good fitness, and the negative Q^2^ from the 200-time permutation tests suggested no overfitting in OPLS-DA models (Supplementary Table S1). At the baseline, the differentially expressed metabolites between BH and BL groups, as well as between BM and BL groups, at the baseline were analyzed by using the univariate t-test (predicted by the *p* value and false discovery rate (FDR) corrected *p*) and multivariate OPLS-DA analysis (predicted by the variable importance in the projection (VIP)) (Table 2). Taking the BL group as a reference, 75 differential metabolites (67 upregulated/8 downregulated) in the BH group (Supplementary Table S2) and 100 differential metabolites (92 upregulated/8 downregulated) in the BM group (Supplementary Table S3) were observed in the combination mode of positive and negative ions (FDR corrected *p* < 0.05, VIP > 1.0). A total of 43 metabolites were consistently implicated in these two comparisons (BH vs. BL and BM vs. BL, Table 2), and were preliminarily identified as the metabolic biomarkers responsible for SHS exposure in baseline analyses. Additionally, a heatmap was generated to visually compare the average normalized amount of these 43 differentially expressed metabolites among the three groups (Figure 3).

The color of each section represents the abundance value of metabolite calculated by the relative quantitation normalization method. Each row corresponds to data for a specific metabolite, and each column represents the BL, BM, or BH group. Different colors represent the different intensity levels of metabolites.

### 3.3. Study Subjects at Follow-Up

As shown in Figure 1, of the 51 children at the baseline, 10 children were excluded at 2 months after intervention due to the following: (a) Smokers reported not complying with the intervention (seven children) and (b) children’s cotinine variation was at the boundary value of stratification at follow-up (three children). Therefore, 41 children were included in the longitudinal analysis, with 21 children showing an unchanged cotinine concentration within the range of 0.5 ng/mL and defined as the intervention-no-changed (INC) group, and 20 children showing a declined cotinine concentration of more than 2 ng/mL and defined as the intervention-declined (ID) group.

The follow-up data are presented in Table 1. For family smokers in the ID group, the smoking frequency decreased from 7.00 (SD 0.00) to 3.71 (SD 3.14) (paired Student’s *t*-test, *p* = 0.004), and the smoking amount decreased from 21.38 (SD 11.17) to 12.56 (SD 6.63) (paired Student’s *t*-test, *p* = 0.020). As expected, there was no significant difference in the smoking frequency and smoking amount before and after the intervention in the INC group (*p* > 0.050). For children’s overall health status and respiratory symptoms reported by their caregivers, there was no significant difference before and after the intervention in both the ID and INC groups.

### 3.4. Longitudinal Metabolomics Analysis at Follow-Up

**Figure 4:**
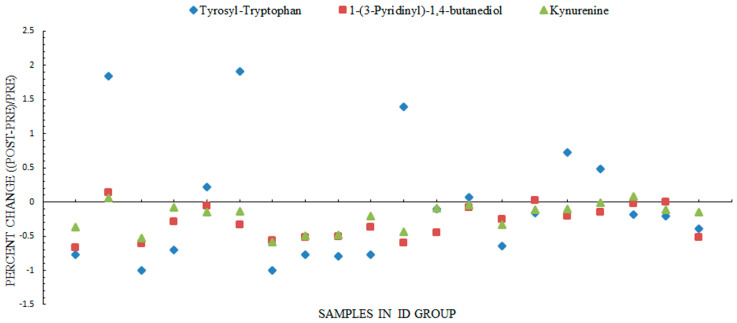
Percent change of the three differential metabolites at post-intervention compared to pre-intervention for each child from the ID group. Blue, tyrosyl-tryptophan; red, 1-(3-pyridinyl)-1,4-butanediol; green, kynurenine; and ID, concentration of nicotine in children’s urine was decreased after the smoking-cessation intervention for smokers, which is called the intervention-declined group (ID) for short.

For the 43 differential metabolites implicated in the cross-sectional analysis, a paired *t*-test was performed to verify the differential urinary metabolites through a comparison of pre-intervention and post-intervention paired-samples from each child in the ID group and INC group, respectively. The metabolites showing significant differences in the ID group (FDR corrected *p* < 0.05) while no change in the INC group (FDR corrected *p* > 0.05) after 2 months of intervention were finally identified as the exact urinary metabolic biomarkers of SHS exposure. Details of the paired comparison for the 43 metabolites are shown in Supplementary Table S4. Table 3 displays the three metabolites confirmed to be related to SHS exposure in the longitudinal analysis, including tyrosyl-tryptophan, 1-(3-pyridinyl)-1,4-butanediol, and kynurenine. The corresponding decreases in the ID group were 0.42-fold for the tyrosyl-tryptophan level (FDR corrected *p* = 0.011 in the ID group and FDR corrected *p* = 1.000 in the INC group), 0.69-fold for the 1-(3-pyridinyl)-1,4-butanediol level (FDR corrected *p* = 0.009 in the ID group and FDR corrected *p* = 1.000 in the INC group), and 0.78-fold for the kynurenine level (FDR corrected *p* = 0.036 in the ID group and FDR corrected *p* = 1.000 in the INC group). Additionally, Figure 4 visually depicts the specific association trend of these three metabolites with the cotinine concentration in each sample of 20 children from the ID group. With the decrease of the cotinine concentration after intervention for smokers, the tyrosyl-tryptophan level declined in 65% (13/20) of samples, 1-(3-pyridinyl)-1,4-butanediol level declined in 90% (18/20) of samples, and kynurenine level declined in 90% (18/20) of samples.

### 3.5. Metabolic Pathway Analysis

**Figure 5:**
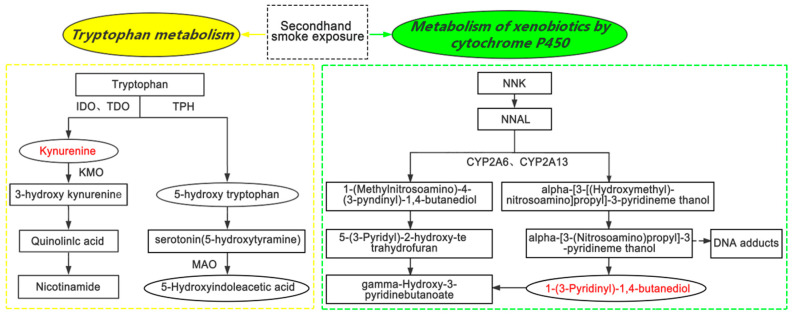
Simplified schematic of the relationship between exposure to SHS and metabolites following the KEGG pathway database.

As shown in Table 4, KEGG pathway enrichment analysis suggested that 1-(3-pyridinyl)-1,4-butanediol was significantly enriched in xenobiotic metabolism by cytochrome P450 (*p* = 0.040), and kynurenine was significantly enriched in tryptophan metabolism (*p* = 0.030). Figure 5 depicts a simplified schematic of the involved metabolism pathway of these two metabolites following the KEGG pathway database.

Red represents the metabolites reported in the present study, which showed a positive relation with SHS exposure. The dotted arrows indicate indirect reactions. Abbreviations: IDO, indoleamine 2,3-dioxygenase; MAO, monoamine oxidase; TDO, tryptophan 2,3-dioxygenase; TPH, tryptophan hydroxylase; KMO, kynurenine 3-monooxygenase; NNK, 4-(methylnitrosamino)-1-(3-pyridyl)-1-butanone; NNAL, 4-(N-nitrosomethylamino)-1-(3-pyridyl)-1-butanol; and CYP, Cytochrome P450.

## 4. Discussion

To the best of our knowledge, this is the first study to combine cross-sectional and longitudinal metabolomics to clarify the SHS-induced changes in urinary metabolites in children. Three metabolic biomarkers, including tyrosyl-tryptophan, 1-(3-pyridinyl)-1,4-butanediol, and kynurenine, were identified as being positively associated with cotinine exposure in children. Among these three urinary metabolites, it is the first time that tyrosyl-tryptophan and 1-(3-pyridinyl)-1,4-butanediol have been reported to be related to SHS exposure.

The results from our study suggested a positive relation between kynurenine and tyrosyl-tryptophan and SHS exposure. The kynurenine pathway is responsible for tryptophan metabolism, and 95% of tryptophan is metabolized via the kynurenine pathway [38]. Therefore, our results suggest a pivotal relationship between tryptophan/kynurenine metabolism and children’s exposure to SHS. A previous study including 20 prospective cohorts from the US, Europe, Australia, and Asia showed that the highest quintiles of serum kynurenine were associated with a 22–31% higher risk of lung cancer compared with the lowest quintiles [39]. Additionally, the downstream metabolites of kynurenine, including 3-hydroxykynurenine (3HK) and quinolinic acid, are potently neurotoxic and attributed to major neurodegenerative diseases, such as schizophrenia, Alzheimer’s disease, Huntington’s disease, bipolar disorder, and depression [40,41,42]. Such an adverse role of kynurenine is furthered here by implicating its positive association with children’s exposure to SHS in our study, indicating the potential risk of future disease in children exposed to SHS for a long period of time. Oades et al. [43] explored whether the levels of cytokines and tryptophan metabolites were associated with features of the index pregnancy of potential etiological significance and found that increased maternal smoking during pregnancy was associated with decreasing kynurenine levels in attention-deficit hyperactivity disorder (ADHD) children, but increasing kynurenine levels in controls, which is consistent with our findings. However, there are also studies showing the opposite conclusion. Naz et al. found that in chronic obstructive pulmonary disease (COPD) patients, the level of kynurenine, which is the main product of tryptophan [44], decreased in smokers relative to never-smokers [38]. Mathai et al. [45] reported that in schizophrenic patients, current smokers showed lower kynurenine levels than past smokers, which further elucidates the neurobiological underpinnings of altered kynurenine levels in smokers. In these studies, there were non-significant decreases in tryptophan levels. Therefore, the lower kynurenine in schizophrenic patients may be due to the decreased dietary intake in schizophrenic patients (broadly reported in smokers) [46]. It is supposed that people with specific diseases may show different change patterns in metabolites in reaction to SHS exposure due to factors such as the disease itself [47], diet, medication, physiological status, and so on.

We also found a positive relation between tyrosyl-tryptophan and the cotinine concentration. Tryptophan has been extensively studied, but evidence of tyrosyl-tryptophan is sparse. Tryptophan is a nutritionally essential amino acid for both humans and animals. In addition to acting as a building block for protein synthesis, tryptophan and its metabolites are crucial for maintaining neurological function, immunity, and homeostasis in the body [48]. Paternal smoking during maternal pregnancy was related to increased tryptophan in control children compared to children with ADHD [43].

Our study is the first to reveal a positive correlation between urine 1-(3-pyridinyl)-1,4-butanediol and the cotinine concentration. As shown in Figure 5, 1-(3-pyridinyl)-1,4-butanediol participates in the 4-(*N*-nitrosomethylamino)-1-(3-pyridyl)-1-butanol (NNAL) pathway and serves as a downstream metabolite of NNAL. NNAL is the most potent tobacco-specific carcinogen, and it has been frequently used as a biomarker to assess human smoke exposure [49]. Both urinary cotinine and NNAL are sensitive and specific biomarkers for discriminating the source of tobacco smoke exposure. The half-life of NNAL (10–16 days) is much longer than that of cotinine (16 h), suggesting that NNAL might be a better measurement of tobacco exposure over time and that NNAL might be a better biomarker when sampling cannot be done in temporal proximity to tobacco smoke exposure [50]. Our data from untargeted metabolomics suggested that 1-(3-pyridinyl)-1,4-butanediol may be a supplementary biomarker of NNAL for long-term exposure to SHS.

A previous study on metabolites relevant to children’s exposure to SHS mainly focused on targeted metabolomics. A randomized clinical trial in 195 female smokers with children aged ≤ 10 years residing in their homes aimed to promote smoke-free homes through biomarker feedback documenting a child’s exposure to tobacco toxins, documenting the levels of nicotine, cotinine, and NNAL [51]. A previous study in children living in homes of hookah-only smokers and nonsmokers examined the child uptake of nicotine, the carcinogen 4-(methylnitrosamino)-1-(3-pyridyl)-1-butanone (NNK), and the toxicant acrolein by analyzing their corresponding metabolites cotinine, 4-(methylnitrosamino)-1-(3-pyridyl)-1-butanol (NNAL) and NNAL-glucuronides (total NNAL), and 3-hydroxypropylmercapturic acid, and the results provide evidence for the uptake of nicotine, the tobacco-specific lung carcinogen NNK, and the ciliatoxic and cardiotoxic agent acrolein in children living in homes of hookah smokers compared with nonsmokers [52]. Although recent studies used the untargeted metabolomics method to explore smoking-relevant metabolites, they only focused on active adult smoking. Garcia-Perez et al. applied CE-MS to a metabolomics analysis of human urine from cigarette smokers and nonsmokers and detected significant changes in urinary glycine and serine, which are intermediates in the metabolism of glutathione in cigarette smokers and nonsmokers [53]. Seow et al. performed a prospective study to examine the association between untargeted urinary metabolomics and the risk of lung cancer among women in China and reported that an increased level of urinary 5-methyl-2-furoic acid was associated with a decreased risk of lung cancer [54]. Such an application of untargeted metabolomics is furthered here by implicating urinary metabolomic changes that are associated with children’s exposure to SHS.

Several limitations of this study should be acknowledged. First, although we combined the cross-sectional baseline comparison with the longitudinal pre-intervention and post-intervention comparison to remove the influence of individual differences and improve the reliability of the results, this study included a preliminary analysis with a small sample size, and studies of larger populations with different groups of children across Asia or with different ethnicities would give a better picture of the connection between urinary metabolites and SHS exposure. Second, the urine samples were only collected once, and we were unable to assess the temporal trends of metabolites over time, making it impossible to derive the long-term effects of metabolites on children with SHS exposure. Subsequent studies should measure urine samples multiple times to obtain long-term influence trends of metabolites. Third, although our study suggests tyrosyl-tryptophan, 1-(3-pyridinyl)-1,4-butanediol, and kynurenine as urinary metabolic biomarkers for SHS exposure, which may be involved in the pathogenesis of disease of the respiratory system, further work, including long-term observations of children and animal experiments, will be needed to determine whether these urinary metabolic biomarkers are predictors of long-term illness. Fourthly, the study lacks urine samples from control children, who were not exposed to second-hand smoke, because this study was based on the smoking-cessation intervention program and because it is difficult to obtain preschool children’s samples and information in nonsmoking families. However, we set low, medium, and high urine cotinine levels and treated the low-concentration group as the control group to perform multiple comparison analyses, in order to address this limitation. Fifthly, we did not test the markers of kidney function in urine, which may have some influence on the level of cotinine and metabolites in urine. Further research with elaborate markers of kidney function in urine is needed to confirm the SHS-associated metabolites identified in our study. Finally, although, in metabolomics work, it is common to use a criterion of a 50% rule to retain the metabolites with missing intensity values in no more than 50% of the samples for statistical analysis [33], the imputation of missing data with the 50% criterion may be risky and a comparison of the values with those of other imputation strategies is needed in analysis, such as random forests [55].

## 5. Conclusions

In summary, this study is the first to have combined cross-sectional and longitudinal metabolomics analysis to examine urinary biomarkers in response to children’s SHS exposures. In addition to the well-established nicotine metabolite kynurenine, we newly identified tyrosyl-tryptophan and 1-(3-pyridinyl)-1,4-butanediol as potential metabolic markers and related functional pathways affected by SHS in children, contributing new insights into the pathophysiological mechanism of SHS. Importantly, our findings indicated that individual differences should be taken into account when exploring urinary metabolites related to children’s exposure to SHS.

## Figures and Tables

**Table 1:** Smoking behavior of smokers and the health status of children at the baseline and follow-up.

Baseline Characteristic	Total	Cotinine Concentration			*p* Value ^c^	
		BL (*n* = 17) (>0.5, <2 ng/mL)	BM (*n* = 17) (2~10 ng/mL)	BH (*n* = 17) (>10 ng/mL)		
**Smokers**						
Smoking frequency ^a^, mean ± SD, time	6.88 (0.62)	6.65 (1.03)	7.00 (0.00)	7.00 (0.00)	0.171	
Cigarette amount ^b^, mean ± SD, number	19.70 (10.20)	16.00 (8.64)	20.75 (10.36)	22.41 (10.41)	0.172	
**Children**						
Health condition						
good, *n* (%)	45 (88)	16 (94)	15 (88)	14 (82)	0.567	
bad, *n* (%)	6 (12)	1 (6)	2 (12)	3 (18)		
Throat irritation or pain						
yes, *n* (%)	9 (18)	4 (25)	4 (24)	1 (6)	0.276	
no, *n* (%)	41 (82)	12 (75)	13 (76)	16 (94)		
Wheeze						
yes, *n* (%)	9 (18)	1 (6)	3 (18)	5 (29)	0.223	
no, *n* (%)	41 (82)	15 (94)	14 (82)	12 (71)		
Nasal obstruction						
yes, *n* (%)	12 (24)	2 (12)	5 (29)	5 (29)	0.375	
no, *n* (%)	39 (76)	15 (88)	12 (71)	12 (71)		
**Follow-up characteristic**	**ID (*n* = 20) (changed cotinine concentration: ≥2 ng/mL)**			**INC (*n* = 21) (changed cotinine concentration: ±0.5 ng/mL)**		
	**Pre-intervention**	**Post-intervention**	***p* value**	**Pre-intervention**	**Post-intervention**	***p* value**
**Smokers**						
Smoking frequency, mean ± SD, time	7.00 (0.00)	3.71 (3.14)	0.004 ^d^	6.88 (0.48)	6.59 (0.84)	0.082 ^d^
Smoking amount, mean ± SD, number	21.38 (11.17)	12.56 (6.63)	0.020 ^d^	17.31 (9.02)	17.24 (8.11)	0.276 ^d^
**Children**						
Health condition						
good, *n* (%)	19 (95)	20 (100)	1.000 ^e^	20 (95)	19 (90)	1.000 ^e^
bad, *n* (%)	1 (5)	0 (0)		1 (5)	2 (10)	
Throat irritation or pain						
yes, *n* (%)	2 (10)	1 (6)	1.000 ^e^	4 (19)	0 (0)	0.125 ^e^
no, *n* (%)	18 (90)	19 (94)		17 (81)	21 (100)	
Wheeze						
yes, *n* (%)	4 (20)	0 (0)	0.125 ^e^	0 (0)	0 (0)	/
no, *n* (%)	16 (80)	20 (100)		21 (100)	21 (100)		
Nasal obstruction						
yes, *n* (%)	4 (20)	0 (0)	0.125 ^e^	3 (14)	1 (6)	0.500 ^e^
no, *n* (%)	16 (80)	20 (100)		18 (86)	20 (94)	

SD, standard deviation; BL, low-level secondhand smoke (SHS) exposure at the baseline; BM, medium-level SHS exposure at the baseline; BH: high-level SHS exposure at the baseline; ID group, intervention-declined group; INC, intervention-no-changed group; SD, standard deviation; ^a^ days of smoking in the past week; ^b^ the average daily cigarette amount in the past week; ^c^ analysis of variance (ANOVA) for continuous variables and Fisher’s exact test for categorical variables; ^d^ *p* value of the paired Student’s *t*-test; and ^e^ *p* value of McNemar’s test.

**Table 2:** Differential urinary metabolites at different levels of SHS exposure in children at the baseline.

Metabolites	BH vs. BL				BM vs. BL			
	VIP	FC	*p* Value	FDR *p*	VIP	FC	*p* Value	FDR *p*
**Peptides**								
Valyl-Phenylalanine	1.83	1.32	<0.001	0.010	1.92	1.39	<0.001	0.011
Ile Asn Asp	1.82	1.45	<0.001	0.010	1.52	1.44	0.003	0.016
Tyrosyl-Tryptophan	1.74	2.00	0.002	0.023	1.67	1.99	0.007	0.026
Arg Val Asp Gly	1.74	1.49	<0.001	0.011	1.64	1.49	<0.001	0.011
Asn Glu Val	1.69	1.62	0.009	0.042	1.70	1.68	0.002	0.015
Phe Leu Gly	1.64	1.62	0.001	0.018	1.46	1.53	0.004	0.018
Hexanoylglycine	1.61	1.46	<0.001	0.013	1.15	1.43	0.002	0.014
Asp Ile Glu	1.54	1.49	0.004	0.028	1.52	1.46	0.003	0.017
Isonicotinylglycine	1.37	1.11	0.003	0.028	1.28	1.10	0.012	0.036
Gamma-Glu-Leu	1.35	1.17	0.004	0.029	1.31	1.19	0.003	0.017
(2S)-2-{[1-(*R*)-Carboxyethyl]amino}pentanoate	1.34	1.39	0.012	0.050	1.37	1.42	0.006	0.023
Asp Gly Pro	1.33	1.17	0.003	0.026	1.22	1.15	0.018	0.044
Gly Asp Tyr	1.27	1.22	0.007	0.038	1.44	1.24	0.004	0.018
*N*-Acetylaspartylglutamic acid	1.26	1.11	0.001	0.020	1.19	1.10	0.003	0.015
Hydroxyprolyl-Valine	1.25	1.15	0.004	0.031	1.24	1.13	0.005	0.022
Asp Cys Arg	1.20	1.22	0.007	0.037	1.33	1.22	0.003	0.016
Glu Val	1.16	1.12	0.010	0.044	1.23	1.13	0.006	0.023
DL-o-Tyrosine	1.16	1.49	0.012	0.050	1.41	1.65	0.002	0.014
**Lipids**								
LPA(P-16:0e/0:0)	1.97	1.58	0.002	0.024	1.58	1.55	0.009	0.030
20-Hydroxy-leukotriene E4	1.65	1.33	0.001	0.015	1.77	1.38	<0.001	0.011
Avocadyne 2-acetate	1.64	1.61	0.003	0.026	1.53	1.55	0.011	0.032
11-Hydroxyprogesterone 11-glucuronide	1.52	1.30	0.007	0.037	1.51	1.30	0.015	0.040
17-HYDROXYPROGESTERONE	1.47	1.27	<0.001	0.009	1.28	1.23	0.001	0.013
(±)-Octanoylcarnitine	1.45	1.23	0.004	0.028	1.29	1.20	0.007	0.026
**Carbohydrates**								
2,8-Dihydroxyquinoline-beta-d-glucuronide	2.01	1.57	0.008	0.040	1.39	1.52	0.007	0.025
5-Megastigmen-7-yne-3,9-diol 3-glucoside	1.56	1.52	0.001	0.019	1.43	1.46	0.003	0.016
**Nucleosides**								
Deoxyadenosine	1.47	1.47	0.004	0.029	1.63	1.56	0.003	0.016
N4-Acetylcytidine	1.24	1.15	0.004	0.031	1.23	1.15	0.003	0.016
N6-Carbamoyl-l-threonyladenosine	1.19	1.11	0.008	0.040	1.44	1.13	0.002	0.014
cAMP	1.04	1.13	0.012	0.050	1.17	1.13	0.006	0.022
**Indoles**								
Indole-3-acetamide	1.35	1.26	0.002	0.025	1.07	1.22	0.008	0.027
Indoleacrylic acid	1.23	1.11	0.005	0.034	1.44	1.13	0.002	0.014
5-Hydroxy-l-tryptophan	1.23	1.12	0.002	0.023	1.28	1.12	0.002	0.014
5-Hydroxyindoleacetic acid	1.07	1.15	0.009	0.043	1.21	1.11	0.017	0.042
**Carbonyl compounds**								
Kynurenine	1.50	1.28	0.001	0.014	1.41	1.28	0.002	0.014
**Pyridines**								
1-(3-Pyridinyl)-1,4-butanediol	1.64	1.35	0.003	0.028	1.29	1.31	0.004	0.018
**Piperidines**								
Methyprylon	1.55	1.70	0.006	0.037	1.17	1.79	0.002	0.014
**Flavonoid glycosides**								
Kaempferol 3-rhamnoside 7-xyloside	1.36	1.50	0.001	0.041	1.39	1.60	0.001	0.043
**Amines**								
1-Methylhistamine	1.33	1.29	0.001	0.018	1.24	1.36	0.001	0.012
**Alcohols**								
Pantothenic Acid	1.24	1.11	0.003	0.027	1.39	1.10	0.002	0.014
**Not known**								
Dimethylmaleic acid anhydride	1.39	1.24	0.002	0.023	1.31	1.24	0.004	0.018
(*R*)-(+)-2-Pyrrolidone-5-carboxylic acid	1.08	1.08	0.003	0.028	1.00	1.07	0.003	0.016
4-formyl Indole	1.04	1.16	0.007	0.038	1.15	1.13	0.014	0.038

BH, high-level SHS exposure at the baseline; BM, medium-level SHS exposure at the baseline; BL, low-level SHS exposure at the baseline; VIP, variable importance in the projection scores; FC (BH/BL), fold change, as determined by average relative quantitation obtained from the BH group/BL group, where a value of less than 1 indicates a decrease in the metabolites of group BH; FC(BM/BL), fold change, as determined by average relative quantitation obtained from the BM group/BL group, where a value of less than 1 indicates a decrease in the metabolites of group BM; and FDR *p*, false discovery rate corrected *p* value.

**Table 3:** Paired *t*-test for final differential urinary metabolites conducted through a comparison of pre-intervention and post-intervention paired-samples from each child in the ID group and INC group, respectively.

Metabolites	ID Group			INC Group		
	FC (Post/Pre)	*p* Value	FDR *p*	FC (Post/Pre)	*p* Value	FDR *p*
Tyrosyl-Tryptophan	0.42	<0.001	0.011	1.26	0.174	1.000
1-(3-Pyridinyl)-1,4-butanediol	0.69	<0.001	0.009	1.02	0.853	1.000
Kynurenine	0.78	<0.001	0.036	1.02	0.694	1.000

ID group, intervention-declined group; INC, intervention-no-changed group; FC, fold change; and FDR *p*, false discovery rate corrected *p* value.

**Table 4:** The metabolic pathways for identified urinary metabolic biomarkers.

Metabolite	Molecular Formula	Library ID ^a^	Related Pathway	*p* Value ^b^
Tyrosyl-Tryptophan	C20H21N3O4	HMDB0029116	Not Known	-
1-(3-Pyridinyl)-1,4-butanediol	C9H13NO2	HMDB0062266	Metabolism of xenobiotics by cytochrome P450	0.040
Kynurenine	C10H12N2O3	HMDB0000684	Tryptophan metabolism	0.030

^a^ Identity from the human metabolite database (HMDB) database. ^b^ *p* value calculated from the Kyoto Encyclopedia of Genes and Genomes (KEGG) pathway enrichment analysis. Criteria: *p* value < 0.05 was defined as significantly enriched.

## Data Availability

The data that support the findings of this study are available from the corresponding author upon reasonable request. Restrictions apply to public availability of these data due to confidential agreement included in the consent form.

## Supplementary Materials

The following are available online at https://www.mdpi.com/1660-4601/18/2/710/s1, Table S1: Model validation and permutation results for OPLS-DA models, Table S2: Metabolites with statistically significant differences between baseline high with baseline low group, Table S3: Metabolites with statistically significant differences between baseline medium with baseline low group, Table S4: Paired t-test to verify 43 urinary metabolites discovered at baseline through the comparison between pre-intervention and post-intervention paired-samples from each child at ID group and INC group respectively.
