# Supplementary material for: Untargeted Urinary Metabolomics and Children’s Exposure to Secondhand Smoke: The Influence of Individual Differences
Source: Int J Environ Res Public Health. 2021 Jan 15;18(2):710. doi: 10.3390/ijerph18020710 (PMC7830063; doi:10.3390/ijerph18020710)
Supplement: Supplementary file 1 [file ijerph-18-00710-s001.pdf]

Supplementary Table S1. Model validation and permutation results for OPLS-DA models.

	7-fold cross-validation				200-time permutation			
	BH VS BL		BM VS BL		BH VS BL		BM VS BL	
	R^2Y	Q^2	R^2Y	Q^2	R^2Y	Q^2	R^2Y	Q^2
**Positive-ion mode**	0.94	0.44	0.76	0.03	0.92	-0.28	0.73	-0.42
**Negtive-ion mode**	0.85	0.17	0.76	-0.12	0.71	-0.35	0.71	-0.12

BL: low level SHS exposure at baseline. BM: medium level SHS exposure at baseline. BH: high level SHS exposure at baseline.

Supplementary Table S2. Metabolites with statistically significant differences between baseline high with baseline low group.

Metabolites	BH VS BL			
	VIP	FC(BH/BL)	*P* value	FDR *P*
**Peptides**				
Valyl-Phenylalanine	1.83	1.32	<0.001	0.010
Ile Asn Asp	1.82	1.45	<0.001	0.010
Tyrosyl-Tryptophan	1.74	2.00	0.002	0.023
Arg Val Asp Gly	1.74	1.49	<0.001	0.011
Asn Glu Val	1.69	1.62	0.009	0.042
Phe Leu Gly	1.64	1.62	0.001	0.018
Hexanoylglycine	1.61	1.46	<0.001	0.013
Asp Ile Glu	1.54	1.49	0.004	0.028
Isonicotinylglycine	1.37	1.11	0.003	0.028
Gamma-Glu-Leu	1.35	1.17	0.004	0.029
(2S)-2-{[1-(R)-Carboxyethyl]amino}pentanoate	1.34	1.39	0.012	0.050
Asp Gly Pro	1.33	1.17	0.003	0.026
Gly Asp Tyr	1.27	1.22	0.007	0.038
N-Acetylaspartylglutamic acid	1.26	1.11	0.001	0.020
Hydroxyprolyl-Valine	1.25	1.15	0.004	0.031
Asp Cys Arg	1.20	1.22	0.007	0.037
Glu Val	1.16	1.12	0.010	0.044
DL-o-Tyrosine	1.16	1.49	0.012	0.050
Asp Phe	1.10	1.10	0.007	0.037
Ser His Ala Gln	1.08	0.81	0.009	0.043
Phe Leu Lys Phe	1.12	0.91	<0.001	0.010
N-Methylglutamic acid	1.20	1.14	0.002	0.025
Agaritinal	1.26	2.80	0.003	0.028
Asp Ile Thr	1.33	1.53	0.012	0.050
Asp-Phe	1.41	1.17	0.007	0.037
Hypoglycin	1.42	1.19	0.004	0.030
Galactosylhydroxylysine	1.68	1.39	0.002	0.025
N-Acetylcystathionine	1.68	1.56	0.005	0.034
L-beta-aspartyl-L-leucine	1.45	1.17	0.008	0.039
**Lipids**				
LPA(P-16:0e/0:0)	1.97	1.58	0.002	0.024
20-Hydroxy-leukotriene E4	1.65	1.33	0.001	0.015
Avocadyne 2-acetate	1.64	1.61	0.003	0.026
11-Hydroxyprogesterone 11-glucuronide	1.52	1.30	0.007	0.037
17-HYDROXYPROGESTERONE	1.47	1.27	<0.001	0.009
(±)-Octanoylcarnitine	1.45	1.23	0.004	0.028
PE(14:0/22:4(7Z,10Z,13Z,16Z))	1.08	0.87	0.002	0.024
Psychosine sulfate	1.27	2.03	0.006	0.035

**Carbohydrates**				
2,8-Dihydroxyquinoline-beta-D-glucuronide	2.01	1.57	0.008	0.040
5-Megastigmen-7-yne-3,9-diol 3-glucoside	1.56	1.52	0.001	0.019
Clitocine	1.48	1.96	0.012	0.050
N-Acetylneuraminic acid	1.72	1.27	0.005	0.031
**Nucleosides**				
Deoxyadenosine	1.47	1.47	0.004	0.029
N4-Acetylcytidine	1.24	1.15	0.004	0.031
N6-Carbamoyl-L-threonyladenosine	1.19	1.11	0.008	0.040
cAMP	1.04	1.13	0.012	0.050
**Indoles**				
Indole-3-acetamide	1.35	1.26	0.002	0.025
Indoleacrylic acid	1.23	1.11	0.005	0.034
5-Hydroxy-L-tryptophan	1.23	1.12	0.002	0.023
5-Hydroxyindoleacetic acid	1.07	1.15	0.009	0.043
**Carbonyl compounds**				
Kynurenine	1.50	1.28	0.001	0.014
2'-Hydroxyacetophenone	1.50	1.43	0.001	0.050
**Pyridines**				
1-(3-Pyridinyl)-1,4-butanediol	1.64	1.35	0.003	0.028
**Piperidines**				
Methyprylon	1.55	1.70	0.006	0.037
**Flavonoid glycosides**				
Kaempferol 3-rhamnoside 7-xyloside	1.36	1.50	0.001	0.041
**Amines**				
1-Methylhistamine	1.33	1.29	0.001	0.018
**Alcohols**				
Pantothenic Acid	1.24	1.11	0.003	0.027
Jasmolone	1.39	1.29	0.003	0.028
**Benzenoids**				
Vanillin	1.43	1.40	0.012	0.050
1-Phenylethyl formate	1.12	1.25	0.008	0.040
Cinnamyl formate	1.01	0.91	0.004	0.031
**Pterins**				
Folinic acid	1.51	1.39	0.003	0.028
**Keto acids**				
2-Amino-5-oxohexanoate	1.38	1.28	0.006	0.037
**Benzodioxoles**				
3,4-Methylenedioxyamphetamine	1.26	1.19	0.001	0.018
**Benzazepines**				
Lorcaserin sulfamate	1.15	0.87	0.001	0.018
**Coumarans**				
2,3-dihydrobenzofuran	1.03	1.10	0.001	0.017
**Not known**				
Dimethylmaleic acid anhydride	1.39	1.24	0.002	0.023
(R)-(+)-2-Pyrrolidone-5-carboxylic acid	1.08	1.08	0.003	0.028
4-formyl Indole	1.04	1.16	0.007	0.038
DL-3,4-Dihydroxyphenyl glycol	1.40	1.41	0.006	0.036
Spisulosine	1.28	0.92	<0.001	0.004
Codonopsine	1.18	1.41	0.011	0.048

N,N-dimethyl-Safingol	1.17	0.94	<0.001	0.006
δ-Valerolactam	1.05	1.09	0.010	0.044
(+)-3,7(11)-Acoradiene	1.02	0.93	<0.001	0.011

BH: high level SHS exposure at baseline. BL: low level SHS exposure at baseline. VIP: variance importance in the projection scores. FC (BH/BL): fold change, as determined by average relative quantitation obtained from BH group / BL group; a value less than 1 indicates a decrease in the metabolites of group BH. FDR *P*: false discovery rate corrected *P* value.

Supplementary Table S3. Metabolites with statistically significant differences between baseline medium with baseline low group.

Metabolites	BM VS BL			
	VIP	FC(BM/BL)	*P* value	FDR *P*
**Peptides**				
Valyl-Phenylalanine	1.92	1.39	<0.001	0.011
Ile Asn Asp	1.52	1.44	0.003	0.016
Tyrosyl-Tryptophan	1.67	1.99	0.007	0.026
Arg Val Asp Gly	1.64	1.49	<0.001	0.011
Asn Glu Val	1.70	1.68	0.002	0.015
Phe Leu Gly	1.46	1.53	0.004	0.018
Hexanoylglycine	1.15	1.43	0.002	0.014
Asp Ile Glu	1.52	1.46	0.003	0.017
Isonicotinylglycine	1.28	1.10	0.012	0.036
Gamma-Glu-Leu	1.31	1.19	0.003	0.017
(2S)-2-{[1-(R)-Carboxyethyl]amino}pentanoate	1.37	1.42	0.006	0.023
Asp Gly Pro	1.22	1.15	0.018	0.044
Gly Asp Tyr	1.44	1.24	0.004	0.018
N-Acetylaspartylglutamic acid	1.19	1.10	0.003	0.015
Hydroxyprolyl-Valine	1.24	1.13	0.005	0.022
Asp Cys Arg	1.33	1.22	0.003	0.016
Glu Val	1.23	1.13	0.006	0.023
DL-o-Tyrosine	1.41	1.65	0.002	0.014
Asn Leu Tyr Thr	1.72	1.48	0.011	0.032
Pro His Cys	1.65	1.43	0.005	0.020
N-Acetyl-L-phenylalanine	1.51	1.49	<0.001	0.011
Cys Val His Lys	1.43	1.60	0.015	0.040
Acetyl-DL-Leucine	1.42	1.37	0.016	0.041
L-Pyridosine	1.36	1.39	0.003	0.017
2-Aminomuconic acid semialdehyde	1.18	1.55	0.019	0.046
DL-2-Aminooctanoic acid	1.16	1.28	0.016	0.041
Leu-Thr-OH	1.00	0.87	0.003	0.016
**Lipids**				
LPA(P-16:0e/0:0)	1.58	1.55	0.009	0.030
20-Hydroxy-leukotriene E4	1.77	1.38	<0.001	0.011
Avocadyne 2-acetate	1.53	1.55	0.011	0.032
11-Hydroxyprogesterone 11-glucuronide	1.51	1.30	0.015	0.040
17-HYDROXYPROGESTERONE	1.28	1.23	0.001	0.013
(±)-Octanoylcarnitine	1.29	1.20	0.007	0.026
(R)-1-O-b-D-glucopyranosyl-1,3-octanediol	1.76	1.42	0.001	0.012
D-Linalool 3-glucoside	1.67	1.88	0.003	0.017
2,6 Dimethylheptanoyl carnitine	1.66	1.12	0.006	0.023
Suberic acid	1.51	1.26	0.005	0.022
(Z)-4-Hepten-1-ol	1.44	1.82	0.002	0.014

2-Methylbutyroylcarnitine	1.40	1.14	0.008	0.027
3,4-Methylenesebacic acid	1.33	1.33	0.008	0.027
Lentialexin	1.32	1.32	0.002	0.014
8-Deoxy-11,13-dihydroxygrosheimin	1.27	1.59	0.012	0.036
Nonate	1.25	1.14	0.005	0.022
Tanavulgarol	1.15	0.86	0.001	0.013
Ethyl 3-phenylpropanoate	1.02	0.90	0.001	0.013
**Carbohydrates**				
2,8-Dihydroxyquinoline-beta-D-glucuronide	1.39	1.52	0.007	0.025
5-Megastigmen-7-yne-3,9-diol 3-glucoside	1.43	1.46	0.003	0.016
Benzoyl glucuronide (Benzoic acid)	1.06	1.48	0.004	0.019
**Nucleosides**				
Deoxyadenosine	1.63	1.56	0.003	0.016
N4-Acetylcytidine	1.23	1.15	0.003	0.016
N6-Carbamoyl-L-threonyladenosine	1.44	1.13	0.002	0.014
cAMP	1.17	1.13	0.006	0.022
8-Hydroxyguanosine	1.50	1.13	<0.001	0.011
**Indoles**				
Indole-3-acetamide	1.07	1.22	0.008	0.027
Indoleacrylic acid	1.44	1.13	0.002	0.014
5-Hydroxy-L-tryptophan	1.28	1.12	0.002	0.014
5-Hydroxyindoleacetic acid	1.21	1.11	0.017	0.042
**Carbonyl compounds**				
Kynurenine	1.41	1.28	0.002	0.014
(Z)-3-Oxo-2-(2-pentenyl)-1-cyclopenteneacetic acid	1.01	1.28	0.002	0.014
4-(2-Aminophenyl)-2,4-dioxobutanoic acid	1.30	1.14	0.005	0.022
**Pyridines**				
1-(3-Pyridinyl)-1,4-butanediol	1.29	1.31	0.004	0.018
3-Pyridylacetic acid	1.38	2.32	0.010	0.031
**Piperidines**				
Methyprylon	1.17	1.79	0.002	0.014
**Flavonoid glycosides**				
Kaempferol 3-rhamnoside 7-xyloside	1.39	1.60	0.001	0.043
**Amines**				
1-Methylhistamine	1.24	1.36	0.001	0.012
Sphinganine	1.16	0.91	<0.001	0.011
2-Hydroxyphenethylamine	1.35	1.13	0.002	0.014
**Alcohols**				
Pantothenic Acid	1.39	1.10	0.002	0.014
**Benzenoids**				
3-Hydroxyhippuric acid	1.63	1.33	0.002	0.014
Vanillylmandelic acid	1.32	1.19	0.004	0.020
Salicyluric acid	1.13	1.17	0.013	0.037
**Pyrans**				
xi-2,3-Dihydro-3,5-dihydroxy-6-methyl-4H-pyran-4-one	2.35	2.51	0.002	0.014
Maltol propionate	1.19	1.94	0.015	0.039
Erinapyrone B	1.11	1.13	0.017	0.043
**Steroids**				
2-Hydroxyestrone sulfate	2.00	1.62	0.005	0.022
**Carboxylic**				

Citric acid	1.74	1.15	0.003	0.017
**Cinnamic acids**				
ferulic acid	1.46	1.84	0.004	0.049
p-Coumaric acid	1.41	1.44	0.016	0.041
2-Hydroxycinnamic acid	1.17	1.12	0.012	0.036
**Quinolines**				
8-Methoxykynurenate	1.53	1.34	0.008	0.028
**Tetrahydrofurans**				
Riesling acetal	1.50	1.26	0.002	0.044
**Phenylpropanoids**				
Coumarin	1.33	1.46	0.013	0.037
**Benzopyrans**				
δ-CEHC	1.10	1.31	0.014	0.038
**Thioacetals**				
2,2,4,4,6,6-Hexamethyl-1,3,5-trithiane	1.08	0.93	0.001	0.013
**Carboximidic acids**				
N1-Acetylspermidine	1.03	1.07	0.015	0.039
**Purines**				
6-Amino-9H-purine-9-propanoic acid	1.02	1.09	0.007	0.024
**Not known**				
Dimethylmaleic acid anhydride	1.31	1.24	0.004	0.018
(R)-(+)-2-Pyrrolidone-5-carboxylic acid	1.00	1.07	0.003	0.016
4-formyl Indole	1.15	1.13	0.014	0.038
9,15-dioxo-11R-hydroxy-2,3,4,5-tetranor-prostan-1,20-dioic acid	1.68	2.45	0.007	0.025
3,4-Dihydroxyphenylpropanoate	1.60	1.20	0.005	0.050
15(R)-15-methyl Prostaglandin A2	1.51	1.48	0.011	0.034
3,5,7,9,11-dodecapentaenoic acid	1.50	1.37	0.006	0.023
1α-hydroxy-25,26,27-trinorvitamin D3 24-carboxylic acid	1.42	1.87	0.016	0.041
epi-4'-hydroxyjasmonic acid	1.40	1.51	0.005	0.022
W123	1.23	1.49	0.002	0.014
1,8-Diazacyclotetradecane-2,9-dione	1.11	0.94	0.001	0.013
cis-2,3-Dihydroxy-2,3-dihydro-p-cumate	1.09	1.21	0.012	0.034
bk-MDDMA	1.03	0.86	0.002	0.014
C-8 Ceramide	1.01	0.89	0.001	0.013

BM: medium level SHS exposure at baseline. BL: low level SHS exposure at baseline. VIP: variance importance in the projection scores. FC (BM/BL): fold change, as determined by average relative quantitation obtained from BM group / BL group; a value less than 1 indicates a decrease in the metabolites of group BM. FDR *P*: false discovery rate corrected *P* value.

Supplementary Table S4. Paired t-test to verify 43 urinary metabolites discovered at baseline through the comparison between pre-intervention and post-intervention paired-samples from each child at ID group and INC group respectively.

Metabolites	ID group			INC group		
	FC (Post/Pre)	*P* value	FDR *P*	FC (Post/Pre)	*P* value	FDR *P*
**Peptides**						
Valyl-Phenylalanine	0.82	0.003	1.000	0.85	0.056	1.000
Ile Asn Asp	0.64	0.002	0.803	0.98	0.800	1.000
Tyrosyl-Tryptophan	0.42	<0.001	**0.011**	1.26	0.174	1.000
Arg Val Asp Gly	0.66	0.003	1.000	1.04	0.683	1.000
Asn Glu Val	0.59	0.009	1.000	0.87	0.388	1.000
Phe Leu Gly	0.64	0.002	0.599	1.16	0.168	1.000
Hexanoylglycine	0.82	0.026	1.000	1.06	0.536	1.000

Asp Ile Glu	0.67	0.005	1.000	0.87	0.275	1.000
Isonicotinylglycine	0.88	0.006	1.000	0.98	0.427	1.000
Gamma-Glu-Leu	0.81	<0.001	0.059	0.96	0.351	1.000
(2S)-2-{[1-(R)-Carboxy-ethyl]amino}pentanoate	0.65	0.001	0.378	0.91	0.433	1.000
Asp Gly Pro	0.86	0.012	1.000	1.02	0.699	1.000
Gly Asp Tyr	0.72	<0.001	0.142	0.97	0.562	1.000
N-Acetylaspartylglutamic acid	0.91	0.016	1.000	1.00	0.897	1.000
Hydroxyprolyl-Valine	0.85	0.003	1.000	1.01	0.892	1.000
Asp Cys Arg	0.77	0.003	0.987	0.96	0.551	1.000
Glu Val	0.82	0.001	0.252	0.97	0.456	1.000
DL-o-Tyrosine	0.63	<0.001	0.112	0.89	0.430	1.000
**Lipids**						
LPA(P-16:0e/0:0)	0.74	0.026	1.000	0.99	0.911	1.000
20-Hydroxy-leukotriene E4	0.71	0.006	1.000	1.07	0.410	1.000
Avocadyne 2-acetate	0.55	0.001	0.481	1.05	0.739	1.000
11-Hydroxyprogesterone 11-glucuronide	0.68	0.001	0.252	1.00	0.991	1.000
17-HYDROXYPROGES-TERONE	0.85	0.033	1.000	1.06	0.195	1.000
(±)-Octanoylcarnitine	0.86	0.024	1.000	1.03	0.549	1.000
**Nucleosides**						
Deoxyadenosine	0.82	0.158	1.000	1.12	0.260	1.000
N4-Acetylcytidine	0.88	0.010	1.000	1.05	0.447	1.000
N6-Carbamoyl-L-threo-nyladenosine	0.85	0.001	0.258	0.99	0.791	1.000
cAMP	0.86	0.004	1.000	0.99	0.844	1.000
**Carbohydrates**						
2,8-Dihydroxyquinoline-beta-D-glucuronide	0.95	0.633	1.000	1.19	0.187	1.000
5-Megastigmen-7-yne-3,9-diol 3-glucoside	0.70	0.004	1.000	0.98	0.872	1.000
**Indoles**						
Indole-3-acetamide	0.80	0.004	1.000	1.05	0.449	1.000
Indoleacrylic acid	0.86	<0.001	0.180	0.98	0.368	1.000
5-Hydroxy-L-tryptophan	0.87	0.001	0.537	1.00	0.973	1.000
5-Hydroxyindoleacetic acid	0.85	0.012	1.000	0.96	0.333	1.000
**Carbonyl compounds**						
Kynurenine	0.78	<0.001	**0.036**	1.02	0.694	1.000
**Pyridines**						
1-(3-Pyridinyl)-1,4-bu-tanediol	0.69	<0.001	**0.009**	1.02	0.853	1.000
**Piperidines**						
Methyprylon	0.62	0.006	1.000	0.82	0.289	1.000
**Flavonoid glycosides**						
Kaempferol 3-rhamnoside 7-xyloside	0.66	0.001	0.069	1.07	0.560	1.000
**Amines**						
1-Methylhistamine	0.86	0.065	1.000	1.01	0.887	1.000
**Alcohols**						
Pantothenic Acid	0.93	0.010	1.000	1.01	0.640	1.000
**Not known**						
Dimethylmaleic acid an-hydride	0.80	0.002	0.596	0.99	0.797	1.000
(R)-(+)-2-Pyrrolidone-5-carboxylic acid	0.92	0.010	1.000	1.00	0.939	1.000

4-formyl Indole	0.86	0.014	1.000	0.95	0.352	1.000

ID group: intervention-declined group. INC: intervention-no-changed group. FC: fold change. FDR P: false discovery rate corrected P value.
